# Costs of two alternative *Salmonella *control policies in Finnish broiler production

**DOI:** 10.1186/1751-0147-49-35

**Published:** 2007-12-04

**Authors:** Susanna Kangas, Tapani Lyytikäinen, Jukka Peltola, Jukka Ranta, Riitta Maijala

**Affiliations:** 1 Department of Food and Environmental Hygiene, Faculty of Veterinary Medicine, PO Box 66, Agnes Sjöberginkatu 2, FI-00014, University of Helsinki, Finland; 2 Finnish Food Safety Authority Evira, Risk Assessment Unit, Mustialankatu 3, FI-00790 Helsinki, Finland; 3 MTT Economic Research, Agrifood Research Finland, Luutnantintie 13, FI-00410 Helsinki, Finland; 4 European Food Safety Authority, Largo N. Palli 5/a, I-43100 Parma, Italy

## Abstract

**Background:**

Costs and benefits of two *Salmonella *control policies for broiler production were described and compared. The control options were the Zoonosis Directive 92/117/EC and the more intense strategy, the Finnish *Salmonella *Control Programme (FSCP).

**Methods:**

The comparison included the *Salmonella *control costs in primary and secondary production and the direct and indirect losses due to *Salmonella *infections in humans in 2000.

**Results:**

The total annual costs of the FSCP were calculated to be 990 400 EUR (0.02 €/kg broiler meat). The average control costs in the broiler production chain were seven times higher with the FSCP than with the Zoonosis Directive alone. However, the public health costs were 33 times higher with the Zoonosis Directive alone. The value of one prevented loss of life per year exceeded the annual control costs of the FSCP.

**Conclusion:**

Due to significant savings in public health costs compared to costs of FSCP, the FSCP was found to be economically feasible.

## Background

Salmonellosis is one of the most commonly reported zoonotic diseases in humans in Europe [1]. In 2000, a total of 105 542 cases were reported in the EU Member States and Norway [2]. However, underreporting of intestinal infections may lead to an underestimation of the true number of cases [[3-5] and [6]]. Human *Salmonella *infections are compulsory notifiable in Finland. In 2000, a total of 2624 human *Salmonella *cases were reported in Finland. The incidence was thus 51/100 000 inhabitants. Of these cases, about 80% were of foreign origin [7].

To prevent foodborne salmonellosis, various control strategies have been designed in different countries. In Finland, *Salmonella *in animals and feedstuffs has been controlled by legislation for decades. In 1990–1994, the annual prevalence in commercial broiler flocks was 0.5–2.9% [8]. In 1995, when Finland became a member of the European Union, the Finnish *Salmonella *Control Programme (FSCP) [9] was established based on the low *Salmonella *prevalence in domestic livestock production. The FSCP was accepted by the European Commission (EC) (European Commission Decision (94/968/EC) approving the operational programme for the control of *Salmonella *in certain living animals and animal products presented by Finland) [10], and it forms the basis for the additional guarantees for importing eggs and meat granted to Finland by the EC. In 2000, 2669 commercial broiler flocks and slightly over 44 million broilers and broiler breeders were slaughtered in Finland. Domestic broiler meat production was 57.4 million kg, and total broiler meat consumption was 2 million kg higher [11]. Only a small proportion of broiler consumed was of foreign origin, and the Finnish broiler meat is hardly ever exported due to the high price of the product. In 2000, 2140 kg of poultry meat was exported and 3 million kg was imported [12].

The objective of the FSCP is to protect consumers by ensuring that *Salmonella *prevalence remains below 1% in swine, bovine and poultry production as well as in meat and eggs derived from these animals [13]. These objectives have been well attained. The FSCP regulates bacteriological investigations for *Salmonella *in primary and secondary production and interventions after detection of any *Salmonella *serovar. Governmental compensations are not paid. However, most farms have voluntary insurance for *Salmonella *infections. The costs of the entire FSCP have been previously reported by Maijala [14] and Maijala & Peltola [15].

Between 1993 and 2004, Council Directive 92/117/EC [16] had set the minimum level for *Salmonella *control in poultry within the European Union. The directive outlines measures for protection against specified zoonoses and specific zoonotic agents in products of animal origin in order to prevent outbreaks of foodborne infections and intoxications. Henceforth in this paper, this directive will be referred to as the Zoonosis Directive. The FSCP was created to fulfil the demands of the Community Zoonosis legislation, which includes the Zoonosis Directive. In 2004, the Zoonosis Directive was replaced by Directive 2003/99/EC of the European Parliament and of the Council on the monitoring of zoonoses and zoonotic agents [17] and by Regulation 2160/2003/EC of the European Parliament and of the Council on the control of salmonella and other specified foodborne zoonotic agents [18]. Directive 2003/99/EC provides instruction on data collection of *Salmonella *in feedingstuffs, food and animals. Its aim is to improve and harmonize data collection on zoonoses in EU Member States. Regulation 2160/2003/EC specifies that Member States must establish a monitoring programme for *Salmonella *serovars with public health significance. Compared with the Zoonosis Directive, the minimum sampling requirements for broiler breeders are similar, with the addition of testing birds before slaughter. The results must be known before transportation to the slaughterhouse. In 2006, a common target for *Salmonella *in all Member States will be established for commercial broiler flocks. Until then, the regulation covers only poultry breeder flocks.

Cost-benefit analysis (CBA) is a simple practical method based on economics that is designed to measure change in welfare due to e.g. a change in government policy or resource use. CBA is commonly used to evaluate societal viability of government policies and regulations and to determine whether a chosen policy provides positive net benefits in a cost-efficient manner [[19] and [20]].

The aim of this study was to compare the costs and benefits of the two *Salmonella *control policies available in Finland, namely the minimum requirements set by Zoonosis Directive 92/117/EEC and the FSCP. The comparison was based on data from 2000.

## Methods

In this analysis, costs of two control options were compared. The first option was the Zoonosis Directive and the second a more intense strategy, the Finnish *Salmonella *Control Programme (FSCP). The difference between these systems was that the Zoonosis Directive set measures only for breeding flocks of poultry and FSCP for all levels of live animal production and also for cutting plants. *Salmonella *Typhimurium and *Salmonella *Enteritidis were the only serovars causing preventive measures by the Zoonosis Directive whereas in the FSCP all *Salmonella *serovars are controlled. In the FSCP meat from positive flocks is heat treated and delivered only in the domestic market. The Zoonosis Directive was chosen as the lower level of salmonella control instead of no control at all since politically it would be the level of control if the FSCP was discontinued. A situation with no salmonella control at all was thus considered an unrealistic alternative. To analyse the costs and benefits of these control policies, the costs were divided into seven subcategories: 1) official *Salmonella *control costs, 2) additional control of primary and secondary production, 3) market disturbances, 4) feed control, 5) additional (not FSCP) control costs to society, 6) public health losses and 7) losses due to premature death. The net benefits of both of the policies were calculated, and the policies were then ranked according to the net benefits. The benefits were at least partly underestimated, as the number of disabilities and the economic consequences of chronic health effects were not included in the analysis. The control costs of the FSCP in primary and secondary production were estimated based on information gathered from broiler-producing companies and FSCP statistics. The main input values are presented in the Additional file 1.

### Control option 1: Zoonosis Directive

The main objective of this directive is to stipulate a reporting system on zoonoses. It also orders monitoring, control and eradication of invasive serovars of *Salmonella *in poultry breeding flocks. According to this directive, faecal specimens from parent-rearing houses are analysed three times per flock. When *Salmonella *Typhimurium or *Salmonella *Enteritidis is suspected, internal organs are also analysed. In hatcheries, chickens are studied for *Salmonella *every two weeks by sampling bottom papers from boxes or meconium. Of the over 2500 serovars of *Salmonella*, only the detection of the two most important ones (*S*. Typhimurium and *S*. Enteritidis) launch eradication measures in breeding flocks. No requirements are set for broilers in meat production.

### Control option 2: Finnish Salmonella Control Programme (FSCP)

The FSCP covers all the requirements of the Zoonosis Directive and also includes some additional requirements, therefore setting higher costs than the Zoonosis Directive to society and the broiler industry of Finland. The FSCP for broilers encompasses breeding and commercial flocks, hatcheries and, unlike the Zoonosis Directive, also poultry meat cutting plants. Breeding flocks are investigated for *Salmonella *from cage bottom paper or meconium samples when the birds are one day old and from faecal samples at four weeks of age and at two weeks prior to entering the laying house. In the laying period, faecal samples are analysed every eighth week. Chickens from every breeding flock are investigated at hatcheries for *Salmonella *every two weeks. Since 2001, official sampling supervised by a veterinarian has been compulsory in hatcheries every eighth week. In 2000, the frequency of official sampling was only once a year. Surface swabs from hatchery structures are also analysed. In the FSCP, any serovar of *Salmonella *is sufficient to launch an intervention.

When any serovar of *Salmonella *is detected in a breeding flock, official restrictions are imposed on the farm. A positive result is confirmed by another sampling. The official restrictions include prohibition of egg and animal delivery. In addition an epidemiological investigation is done to identify the source and possible spread of the infection. Hatching eggs originating from the flock are destroyed. The official restrictions result in slaughter or killing of positive breeding flocks. Restrictions are lifted only after the premises have been emptied and disinfected, and surface swab samples yield test negative [13].

Commercial broiler flocks are studied for *Salmonella *once during the rearing period, one to two weeks prior to slaughter. Results of the analyses must be available before a flock is slaughtered. *Salmonella*-positive flocks go to sanitary slaughter and meat is heat-treated and delivered only to the domestic market. Afterwards, the slaughterhouse and farm premises are thoroughly disinfected. Arrival of a new flock is allowed only when negative results of the environmental samples of the poultry house are available.

In 2000, the FSCP sampling frequency at poultry meat cutting plants was one crushed meat sample per week per plant, with a total of 250 crushed meat samples being analysed. Since 2001, the sampling frequency has been dependent on the magnitude of production, resulting in one sample per day in the largest cutting plants. A positive detection in a cutting plant launches compulsory disinfection and analyses of 59 samples within the following five working days. These 59 samples are taken from meat and the structures of the establishment. A positive finding in one of these induces sampling of a further 59 samples until the premises are proven to be free of infection [13].

### Salmonella control in the food production chain

In Finland, *Salmonella *in feedstuffs has been controlled since more than 40 years by legislation, and feed control is an important basis for the FSCP reaching its targets. *Salmonella *control is compulsory for feed manufacturers. In primary and secondary production, own-checking systems and voluntary measures are also applied to control *Salmonella *[13].

### Simulation model

To compare the costs and benefits of these two control options, the benefit-cost (BC) ratios were calculated by dividing the benefit by the cost. The cost was the difference in official control costs in running the FSCP and the Zoonosis Directive. The benefit was the difference in other losses due to *Salmonella*. A BC ratio of less than one indicates that costs exceed benefits, whereas a BC ratio greater than one indicates that benefits exceed costs. The costs and benefits were estimated by constructing a Monte Carlo simulation model with 20 000 iterations using @RISK^® ^3.52 software (Palisade Inc., USA).

By combining probability density of the BC ratio and the distance of each BC ratio from one, a win-lose ratio was achieved. The win-lose ratio is the product of probability and magnitude of winning compared with that of losing. Winning is realized when BC > 1, and losing when BC < 1. The probability of either win (p(win)) or lose (p(lose)) was determined by calculating the number of iterations when the condition was true and dividing it by the total number of iterations. Magnitude is defined as the average distance of BC from 1 (ad) and was calculated separately for situations of BC > 1 (ad(win)) and BC < 1 (ad(lose)). The win-lose ratio is then [p(win)*ad(win)]/([p(lose)*ad(lose)]. The sum p(win)*ad(win)+p(lose)*ad(lose) equals E(E(distance | outcome)) = E(distance), where "outcome" is the indicator function for the type of event, which is either win (outcome = one) or lose (outcome = zero), and where we denote ad(win) = E(distance | win) and ad(lose) = E(distance | lose), respectively. By distance, we denote the absolute value of the difference BC-1.

To estimate the differences in the number of *Salmonella *cases between control options 1 (Zoonosis Directive) and 2 (FSCP), a quantitative microbiological risk assessment model was built. The two key parameters defining the costs (number of human cases and number of infected flocks) were obtained from the risk assessment model [[21,22] and [23]] for both control options. The distributions of these parameters were incorporated into the economic simulation model and were linked with other variables. In the model, human *Salmonella *cases were classified into four categories: hospitalized patients, outpatients, unreported cases and deaths. Public health losses were calculated to consist of human illness costs and productivity losses during illness. Expectancies in the number of additional disinfections in slaughterhouses, heat treatments of infected meat and withdrawals of infected products from the market were dependent on the number of infected flocks based on industry data. The parameters of the two control options were synchronized; the same variable in both options was associated with the same random number during the same iteration. When this was difficult to achieve due to limitations of the software, a link was created of a rank order correlation function (value = 1) mimicking the true synchronization.

## Results

### Control costs in the broiler production chain

In 2000, 26 of the 2669 commercial broiler flocks slaughtered were found to be positive (serovars *S*. Anatum, *S*. Bardo, *S*. Infantis, *S*. Livingstone, *S*. Tennessee and *S*. Thompson). No positive samples from breeding flocks or from hatcheries were detected. The costs of analysis according to the Zoonosis Directive were 83 000 EUR (101 764 USD). The costs of FSCP analyses in primary production were approximately 308 000 EUR. The disinfection costs in primary production under FSCP were 65 000 EUR, and an additional 53 100 EUR were spent on voluntary *Salmonella *control.

In secondary production, 97% (596 000 EUR) of the total costs of the FSCP arose from freezing and heat treatment of meat from positive flocks. The costs of additional *Salmonella *control, i.e. control measures not included in the FSCP, were 39 000 EUR, and governmental administration of the FSCP was estimated to be 21 000 EUR.

The total costs of the FSCP were 990 400 EUR, of which 38% were derived from primary production, 60% from secondary production and 2% from society. The costs were relatively low in primary production since no positive flocks were detected in breeding flocks or hatcheries. The total costs of the FSCP were estimated to be 0.02 EUR/kg of produced broiler meat.

### Public health losses

Based on the model, the public health losses would have been 324 120 EUR with control option 1 (Zoonosis Directive) when premature deaths were not included. The median cost of a single reported human S*almonella *case was 498 EUR when mortality costs were not included. The total public health losses, deaths included, would have been 1 698 700 EUR. At least one premature loss of life was estimated to occur each year.

The public health losses due to domestic broiler meat-borne *Salmonella *infections with the FSCP were calculated to be 60 680 EUR. According to the risk assessment model [[21] and [22]], the median number of *Salmonella*-induced deaths per year was zero with this control strategy. The saving with the FSCP was thus 1 638 000 EUR.

### Benefit-cost ratio

**Figure 1:**
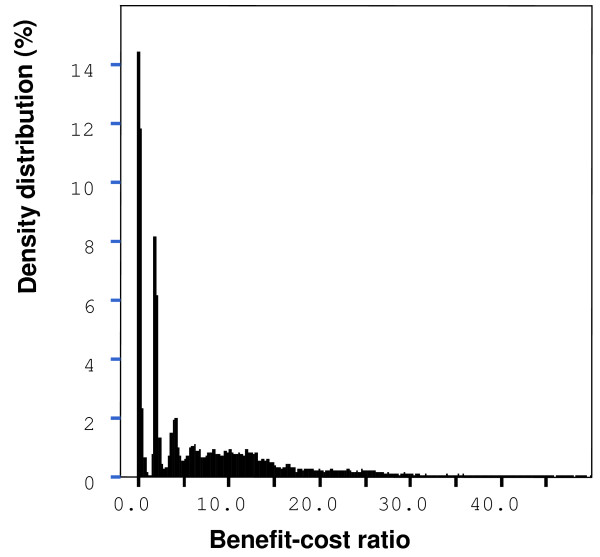
Simulated (20 000 iterations) density distribution of the benefit-cost (BC) ratio of two control options. The effects of *Salmonella*-induced mortality and market disturbances are included in the distribution. Note that 0.84% of the simulated BC values were above the range of the figure (BC ratio > 50.0).

**Figure 2:**
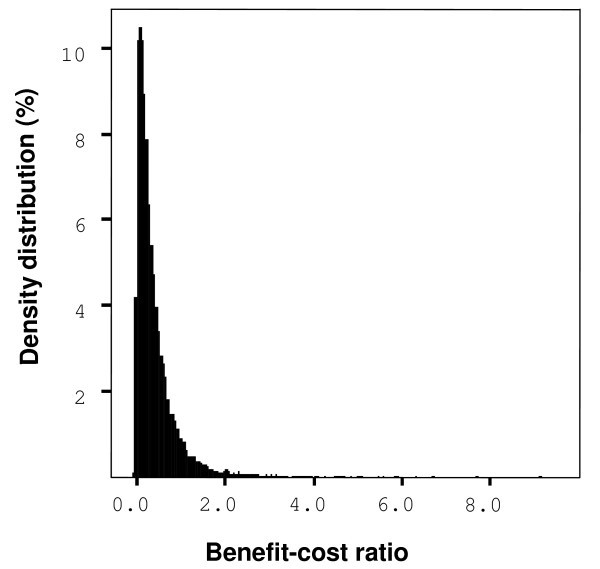
Simulated (20 000 iterations) density distribution of the benefit-cost (BC) ratio of two control options. The effects of *Salmonella*-induced mortality and market disturbances are excluded from the distribution. Note that 0.005% of the simulated BC values were above the range of the figure (BC ratio > 10.0).

The median BC ratio for the FSCP was 4.00 (90% range 0.04–21.25) (Figure 1). This ratio was found to be mainly dependent on the number and costs of recalls. The win-lose ratio describing the probability of winning combined with the magnitude of winning/losing per investments was 28:1 for the FSCP when market disturbances and deaths were included. The median BC ratio was 0.23 (90% range 0.01–0.93, about 29% of the 20 000 iterations were under one) (Figure 2) and the win-lose ratio 0.08:1 when market disturbances and deaths were ignored. The output parameters used to estimate the win-lose ratios between the two control options are presented in Table 1.

**Table 1:** Output parameters used to estimate the win-lose ratio between control option 1 (Zoonoses Directive) and control option 2 (FSCP), including or excluding market disturbances and mortality.

**Assumption**	**Win/Lose**	**Average distance of the BC ratio from 1**	**p**
No market disturbances and no mortality	Win	0.63	0.08
	Lose	0.73	0.92
Market disturbances and mortality	Win	10.08	0.71
	Lose	0.87	0.29

According to sensitivity analysis, the BC ratio was dependent most on the uncertainties in the number of recalls, human epidemics and deaths with the Zoonosis Directive option. The large number of human Salmonella infections in the Zoonosis Directive scenario highlights the benefits of the FSCP. The BC ratio was dependent on several variables that were partially linked to each other. The numbers of deaths and recalls were both related to the number of broiler meat-borne human cases. Therefore, these variables were influenced by several other variables such as number of hospitalized patients, unreported cases and costs due to human epidemics. The BC ratio was strongly dependent on the number of deaths.

## Discussion

Based on this analysis, control costs in the broiler production chain were on average seven times higher when the FSCP was applied than when applying the Zoonosis Directive alone, whereas public health costs were 33 times higher when the Zoonosis Directive alone. Losses due to human infections were mostly dependent on the number of *Salmonella*-induced deaths and their monetary value. Persson & Jendteg [3] and Frenzen et al. [5] have shown that the importance of preventive efforts increases when the estimate for the cost of illness is extended to include a value for reducing the risk of death. In our study, the loss due to one death was 0.95–1.78 (90% range) times higher than the total costs of the FSCP, and thus one prevented death per year makes the FSCP feasible from a societal point of view.

For these types of monetary calculations, it is often difficult to obtain reliable estimations of non-existing situations (here option 1, control based on only the Zoonosis Directive), although they can be the most critical input values used in the calculations. However, in our study, we benefited from the results achieved by the risk assessment model developed to study the public health effects of the FSCP on broiler production [[21] and [23]]. The risk assessment model was used to produce estimations of the number of human cases with these two control options. This gave a better estimate of the health effects of different control options than a deterministic approach or expert opinions, both of which are often used in health impact assessments [24], However, constructing a quantitative risk assessment model is itself a significant workload and therefore may not always be possible due to limited resources.

The role of *Salmonella *as a cause of health problems may have been underestimated in our study. Helms et al. [25] reported in a registry-based study that people with gastrointestinal infections (*Salmonella*, *Campylobacter *and *Yersinia enterocolitica*) had an increased long-term risk of death even after effects of pre-existing illnesses were taken into account. One-year relative mortality among *Salmonella *patients was 2.85 times higher than among matched controls. In addition, *Salmonella *infections may lead to sequelae, including joint and heart problems (e.g. endocarditis, polyarthritis, ankylosing spondylitis and osteomyelitis) [26] and uveitis [27]. Had these long-term effects been taken into consideration, the benefit of the FSCP would probably have been greater. However, not enough data were available to include them in this analysis.

The median cost of reported human *Salmonella *cases was 554 EUR when mortality costs were not included. The median cost of reported cases when deaths were included was 589 EUR. The median cost of all cases was 222 EUR. This difference arises from the number of unreported cases clearly being high and the health costs in this group being lower than in reported cases. When the proportion of deaths is high, the costs per case are higher too. Roberts et al. [6] calculated the average cost per case of infectious intestinal disease to be 253 GBP (358 EUR) and the average cost per *Salmonella *case to be 606 GBP (857 EUR). Mortality costs were not included in that research, but the impact of illness on the ability to carry out normal activities was estimated. Persson & Jendteg [3] estimated the cost per case to be 1200–1500 GBP (1696 – 2120 EUR) when mortality costs were included. In their calculations, mortality was not only considered loss as an economic measure but also value of health per se, i.e. the value of reducing the risk of health loss or death was included. According to the USDA ERS (United States Department of Agriculture, Economic Research Service) Foodborne Illness Cost Calculator [28], the average cost per *Salmonella *case is 2126 USD (1734 EUR) including costs of mortality. ERS uses "willingness-to-pay" (WTP) estimates as the cost of premature death, which was 6.6 million USD (5 384 280 EUR) in 2001.

Based on our calculations, the cost per kilogram of broiler meat, 0.02 EUR/kg, 98% of which was paid by the industry, was relatively low. This result was close to USD 0.02/kg for *Salmonella *control costs in a broiler production chain in Denmark [29]. Although some differences exist in the FSCP and the Danish control programme, they are both based on the principle of top-down eradication of *Salmonella *in the broiler production pyramid.

In the FSCP, the greatest cost to the industry was caused by the heat treatment of meat from positive flocks, whereas for society the greatest cost was due to premature deaths. The significance of heat treatment is affected by the variety of products. In Finland, most broiler meat is sold as fresh, and therefore, the opportunities to use heat-treated broiler meat are limited. For the Finnish broiler industry, the consequences of prohibition of export of meat originating from *Salmonella*-positive flocks has a minor impact since of the amount of exported poultry meat is very small. This study was also based on the assumption that the origin of consumed broiler meat would be the same, i.e. domestic, even if the FSCP were be abandoned and control was based only on the Zoonosis Directive. However, in real life this might not be the case since with removal of additional guarantees agreed by the EC, import from EU and third world countries would probably increase significantly. If this resulted in the replacement of half of the current retail broiler meat by meat with 20–40% contamination, 33–93 times more human cases would be detected compared with the expected value under the current situation [23].

Cost-benefit analysis has been criticized as being mechanistic and unable to account for preferences other than through market prices. Dorfman [30] listed three main shortcomings related to CBA. The first is the difficulty to express complex outcomes through a single money measure. Because of this difficulty, decision-making requires other criteria in addition to money measure as well as a well-functioning democratic process. The second problem is the inability of the CBA to recognize distributional effects, i.e. which societal groups benefit and which suffer from the change in question. The third problem is the uncertainty in results and the difficulty in evaluating the level of this uncertainty. To counteract this drawback, CBA should always be supported by a broad sensitivity analysis. Although these shortcomings may undermine the usability of CBA and, in some cases, cause biased results, Dorfman [30] continues to regard CBA as a good method. Similarly, Randall [31] supports the practical use of CBA. When the analysis considers only one period, discounting is not necessary.

This study shows that the FSCP control policy has been successful. One prevented loss of life covers the control costs in the broiler production chain. The new zoonosis legislation in the EU only slightly augments *Salmonella* control in the broiler production chain compared with the old Zoonosis Directive. Thus, the results presented here also apply to the current situation. EU legislation was renewed in 2003 to improve the prevention and control of zoonoses. *Salmonella *has been identified as a priority target, especially in poultry production. Based on our study, this is reasonable considering the long term public health and economic impact of the societal level. However, the economic efficiency of the FSCP is also based on *Salmonella *control measures, such as feed control, which has been used for decades in Finland. Therefore, the BC ratios would probably be different if these measures were applied in a situation with a higher prevalence of *Salmonella *in broiler production.

## Conclusion

In conclusion, the FSCP for broilers is an economically feasible programme for society compared with the lower level of control provided by the Zoonosis Directive. The FSCP is viable from the poultry meat producer', consumers' and tax payers' points of view.

## List of abbreviations used

BC = benefit-cost

CBA = cost-benefit analysis

FSCP = Finnish *Salmonella *Control Programme

## Competing interests

The author(s) declare that they have no competing interests.

## Authors' contributions

SK, JP and RM were involved in the study design. SK, TL and RM constructed the calculation model in Excel. TL analyzed statistically the data and implemented @risk simulation model. JP was responsible for the economic model. JR and RM were responsible for the risk assessment used as input to this simulation model. SK and RM were responsible for the manuscript preparation. All authors have participated in the manuscript revision and have read and accepted the final manuscript.

## Supplementary Material

Additional File 1 The input variables and values/distributions used in the model.

## References

[B1] Anonymous (2001). Report to the European Commission and to the Council on the measures to be put in force for the control and prevention of zoonoses. http://eur-lex.europa.eu/LexUriServ/site/en/com/2001/com2001_0452en01.pdf.

[B2] SANCO/927/2002, Trends and sources of zoonotic agents in animals, feedstuffs, food and man in the European Union and Norway. European Commission. Health and Consumer Protection Directorate – General.

[B3] Persson U, Jendteg S (1992). The economic impact of poultry-borne salmonellosis: how much should be spent on prophylaxis?. Int J Food Microbiol.

[B4] Baird-Parker AC (1994). Foods and microbiological risks. 1993 Fred Griffith Review Lecture, Delivered at the 126^th ^ordinary meeting of the Society for General Microbiology, 8 September 1993. Microbiology.

[B5] Frenzen P, Riggs TL, Buzby J, Breuer T, Roberts T, Voetch D, Reddy S, the FoodNet Working Group (1999). *Salmonella *cost estimate updated using Food Net data. FoodReview.

[B6] Roberts JA, Cumberland P, Socket PN, Wheeler J, Rodrigues LC, Sethi D, Roderick PJ, on behalf of the infectious intestinal study executive (2003). The study of infectious intestinal disease in England: socio-Economic impact. Epidemiol Infect.

[B7] National Public Health Institute (KTL) (2001). Tartuntataudit Suomessa 2000 (in Finnish) Kansanterveyslaitoksen julkaisuja KTL B 8.

[B8] Maijala R, Ranta J, Seuna E, Peltola J (2005). The efficiency of the Finnish Salmonella Control Programme. Food Contr.

[B9] Ministry of Agriculture and Forestry (23/EEO/2001) *Salmonella *control programme for broilers and turkeys. (In Finnish and Swedish). http://www.mmm.fi/el/laki/d/default.html.

[B10] European Commission Decision (94/968/EC) approving the operational programme for the control of *Salmonella *in certain living animals and animal products presented by Finland. OJ of the European Communities L.

[B11] Poultry Association http://www.siipi.net/siipikarjatuotanto.html.

[B12] (2001). Finnish Food and Drink Industries' Federation.

[B13] Ministry of Agriculture and forestry (2000). Zoonoses in Finland in 1995–1999. Helsinki.

[B14] Maijala R The costs, benefits and effects of the *Salmonella *Control Programme in Finland. Abstract Workshop on Analytical Methods in the Epidemiology of Zoonosis, 23–24th November 1998; Berlin.

[B15] Maijala R, Peltola J Finnish *Salmonella *Control Program – efficiency and viability in food safety promotion. The 10th EAAE Congress; Zaragoga, Spain; 2002, August 28–31.

[B16] Zoonoses Directive named European Council Directive (92/117/EEC) concerning measures for protection against specified zoonoses and specific zoonotic agents in animals and products of animal origin in order to prevent outbreaks of food-borne infections and intoxications, as amended. OJ of the European Communities L.

[B17] European Parliament and the Council Directive (2003/99/EC) on the monitoring of zoonoses and zoonotic agents amending Council Decision 90/424/EEC and repealing Council Directive 92/117/EEC. OJ of the European Communities L.

[B18] European Parliament and Council Regulation (2160/2003) on the control of salmonella and other specified food-borne zoonotic agents. OJ of the European Communities L.

[B19] Dorfman R, Dorfman R, Dorfman NS (1993). An introduction to benefit-cost analysis in economics of the environment – selected readings.

[B20] Krupnick A Valuing health benefits – policy choises and technical issues. RFF Report 2004, March Washington DC.

[B21] Ranta J, Maijala R (2002). A probabilistic transmission model of *Salmonella *in the primary broiler production chain. Risk Anal.

[B22] Maijala R, Ranta J (2004). *Salmonella *in broiler production in Finland – a quantitative risk assessment.

[B23] Maijala R, Ranta J, Seuna E, Pelkonen S, Johansson T (2005). A quantitative risk assessment of the public health impact of the Finnish salmonella control program for broilers. Int J Food Microbiology.

[B24] Veerman JL, Barendregt JJ, Mackenbach JP (2005). Quantitative health impact assessment: current practice and future directions. J Epidemiol Community Health.

[B25] Helms M, Vastrup P, Gerner-Smidt P, Mølbak K (2003). Short and long term mortality associated with foodborne bacterial gastrointestinal infections: registry based study. BMJ.

[B26] Baird-Parker AC (1990). Foodborne salmonellosis. Lancet.

[B27] Saari KM, Vilppula A, Lassus A, Leirisalo M, Saari R (1980). Ocular inflammation in Reiter's disease after Salmonella enteritis. Am J Ophthalmol.

[B28] ERS/USDA Foodborne Illness Cost Calculator. April 15 2003 updated version.

[B29] Wegener HC, Hald T, Wong DLF, Madsen M, Korsgaard H, Bager F, Gerner-Smidt P, Mølbak K (2003). *Salmonella *control programs in Denmark. Emerg Inf Dis.

[B30] Dorfman R (1996). Why benefit-cost analysis is widely disregarded and what to do about it?. Interfaces 26:5 September–October.

[B31] Randall A (1999). Why benefits and costs matter?. CHOICES, Second Quarter.

[B32] Ministry of Agriculture and Forestry (2001). Trends and sources of zoonotic agents in animals, feedingstuffs, food and man in Finland in 2000.

[B33] Ministry of Agriculture and Forestry (2001). Animal diseases and welfare in Finland 2000.

[B34] (2001). Finnish Statistics Office (Tilastokeskus). http://www.tilastokeskus.fi.

[B35] Wheeler J, Sethi D, Cowden J, Wall P, Rodrigues L, Tompkins D, Hudson M, Roderick P (1999). Study of infectious intestinal disease in England: rates in the community, presenting to general practice and reported to national surveillance. BMJ.

[B36] Salomaa J (1993). (In Finnish) Alkoholin käytön haittakustannukset ja verotaso Suomessa. Alko, Alkoholipoliittinen suunnittelu ja tiedotus, Tutkimusseloste No 22.

[B37] National Research and Development Centre for Welfare and Health (STAKES) (2002). Yearbook of alcohol and drug statistics 2001. Helsinki, Finland.

[B38] Roberts JA, Socket PN, Gill ON (1989). Economic impact of a nationwide outbreak of salmonellosis: cost-benefit of early intervention. BMJ.

[B39] Miller SI, Hohmann EL, Pegues DA, Gerald L Mandell, John E Bennet, Raphael Dolin (1995). Mandell, Douglas and Bennetts' principles and practice of infectious diseases.

