# Supplementary material for: Costs of two alternative Salmonella control policies in Finnish broiler production
Source: Acta Vet Scand. 2007 Dec 4;49(1):35. doi: 10.1186/1751-0147-49-35 (PMC2231353; doi:10.1186/1751-0147-49-35)
Supplement: Additional File 1 — The input variables and values/distributions used in the model. [file 1751-0147-49-35-S1.doc]

Additional file 1. The input variables and values/distributions used in the model.

| **Letter in equation** | **Parameter** | **Value or conditional distributions** | **Source of information** |
| --- | --- | --- | --- |
| A | Commercial broiler flocks / year | 2669 | [32] |
| B | Infected flocks with FSCP | 26 | [33] |
|  | Apparent prevalence of *Salmonella* in flocks; FSCP | B / A = 0.97% | [33] |
| C | Infected flocks with Zoonosis Directive | 10 ^(Normal (mean = 2.058 ; sd = 0.268))^ | Simulated; modified distribution from Risk assessment model  [21 and 22] |
|  | Apparent prevalence of *Salmonella* in flocks; Council Directive | C / A Median = 4.27%;  90% range = 1.54% - 11.76% | Simulated; Risk assessment model [21 and 22] |
|  | Direct cost of recall of contaminated products | Not public | Questionnaire 2002 |
|  | Indirect cost of recall of contaminated products | Not public | Questionnaire 2002 |
|  | Inhabitants | 5 171 302 | [34] |
|  | Total number of annually reported human cases | 2 624 | [7] |
| D | Reported broiler meat-borne domestic cases/year; Zoonosis Directive | 10 ^(0.935 * LOG^_10_^(C) + 0.594 + F * 0.253)^  (rounded to the nearest integer) Median = 330; 90% range = 116 - 928 | Simulated; modified distribution from Risk assessment model [21 and 22] |
| E | Reported broiler meat-borne domestic cases/year; FSCP | (7.878 + F * 1.474) ^ 2  (rounded to the nearest integer) Median = 63; 90% range = 36 - 95 | Simulated; modified distribution from Risk assessment model  [21 and 22] |
| F | Random normal distribution for broiler meat-borne domestic cases | Normal (mean = 0 ; sd = 1) |  |
| G | Physician visiting rate | Uniform (min = 0.6; max = 0.73) | Min: Estimation Max: [35] |
|  | Loss due to death (EUR) | Uniform (min = 944 952; max = 1 760 738) | [36 and 37] |
| H | Proportion of reported cases leading to death | Uniform (min = 0.0011; max = 0.0038) | Min: [38]  Max: [5 and 39] |
| I | Hospitalization rate of reported cases | Uniform (min = 0.011; max = 0.04) | Min: [5]  Max: [14] |
| J | Proportion of reported cases of all cases | Beta (alpha 1 = 20; alpha 2 = 80) | [32] |
|  | Deaths; Zoonosis Directive | Poisson (lambda = (H * D)) Median = 1; 90% range = 0 - 4 | Simulated; Risk assessment model [21 and 22] |
|  | Deaths; FSCP | Poisson (lambda = (H * E)) Median = 0; 90% range = 0 - 1 |
|  | Hospitalized; Zoonosis Directive | I * D (rounded to the nearest integer) Median = 8; 90% range = 3 - 25 |
|  | Hospitalized; FSCP | I * E (rounded to the nearest integer) Median = 1; 90% range = 1 - 3 |
|  | Outpatients; Zoonosis Directive | G * D (rounded to the nearest integer) Median = 220; 90% range = 77 - 617 |
|  | Outpatients; FSCP | G * E (rounded to the nearest integer) Median = 41; 90% range = 24 - 64 |
|  | Unreported; Zoonosis Directive | D * (1 / J) - D Median = 1342; 90% range = 448 - 4,006 |
|  | Unreported; FSCP | E * (1 / J) - E Median = 251; 90% range = 133 - 437 |
